# The social–ecological ladder of restoration ambition

**DOI:** 10.1007/s13280-024-02021-8

**Published:** 2024-04-23

**Authors:** Marina Frietsch, Manuel Pacheco-Romero, Vicky M. Temperton, Beth A. Kaplin, Joern Fischer

**Affiliations:** 1 Social-Ecological Systems Institute, School of Sustainability, Leuphana University, Lueneburg, 21335 Lüneburg, Germany; 2 Center of Excellence in Biodiversity and Natural Resource Management, University of Rwanda, Kigali, Rwanda; 3 Department of Biology and Geology, Andalusian Center for the Assessment and Monitoring of Global Change (CAESCG), University of Almería, Almería, Spain; 4 Institute of Ecology, School of Sustainability, Leuphana University Lüneburg, 21335 Lüneburg, Germany; 5 School for the Environment, University of Massachusetts, Boston, MA USA

**Keywords:** Ecosystem restoration, Forest landscape restoration, Grassland restoration, Social–ecological systems

## Abstract

Expanding in both scope and scale, ecosystem restoration needs to embrace complex social–ecological dynamics. To help scientists and practitioners navigate ever new demands on restoration, we propose the “social–ecological ladder of restoration ambition” as a conceptual model to approach dynamically shifting social and ecological restoration goals. The model focuses on three dynamic aspects of restoration, namely degrading processes, restoration goals and remedial actions. As these three change through time, new reinforcing and balancing feedback mechanisms characterize the restoration process. We illustrate our model through case studies in which restoration has become increasingly ambitious through time, namely forest landscape restoration in Rwanda and grassland restoration in Germany. The ladder of restoration ambition offers a new way of applying social–ecological systems thinking to ecosystem restoration. Additionally, it raises awareness of social–ecological trade-offs, power imbalances and conflicting goals in restoration projects, thereby laying an important foundation for finding more practicable and fairer solutions.

## Introduction

With ongoing land degradation, human population growth and anthropogenic climate change, restoration ambitions are rising globally, in both scale and scope. For example, in terms of scale, under the Bonn Challenge, 61 countries have pledged to restore 350 million hectares by 2030 (Dave et al. 2018); the Great Green Wall initiative seeks to restore a 7000-km-long band across the Sahel (Goffner et al. 2019); and the United Nations have declared 2021–2030 the Decade on Ecosystem Restoration (UNEA 2019). In terms of scope, ecosystem restoration evolved from a focus on simply replanting disturbed areas (McDonald 2008), to attention to reference states (Society for Ecological Restoration 2004), the “rewilding” of ecosystems (Perino et al. 2019), and reinstating ecosystem functions and processes (Manning et al. 2018). Today, restoration often pursues diverse social goals such as enhancing intrinsic ecological values, advancing human well-being, supporting livelihoods or empowering local people (Martin 2017). Satisfying the growing ambitions of restoration is challenging, not least because restoration inevitably takes place within a dynamic and contested social–ecological context, in which both biophysical conditions as well as societal priorities and hence expectations of restoration keep shifting (Fischer et al. 2021).

The Society for Ecological Restoration recently proposed restoration standards (Gann et al. 2019) that include a restoration continuum from remediation to ecological restoration, as well as “restoration wheels” to trace restoration progress against various ecological and social goals. Here, we argue that these standards could be complemented by considering how drivers of degradation as well as remedial actions dynamically change in response to evolving needs, values, environmental context, knowledge, policies and resources (see also Keenleyside et al. 2012, p. 53). Integrating such dynamism could help, for instance, to address questions related to restoration in human-created and maintained “novel ecosystems”, such as whether restoring a historical reference state is feasible and desirable. Ecological and ethical challenges posed by ecological novelty are widely acknowledged, but solutions remain controversial (Aronson et al. 2018; Higgs et al. 2018). In addition, legacy effects of past conditions operate both in the ecological (Weidlich et al. 2020) and social spheres (Clay 2019), which further constrains and shapes restoration possibilities and outcomes over time. Perhaps most notably, shifting societal expectations of restoration are rarely considered, although the history of restoration shows that such expectations have changed and hence are likely to continue to change.

In practice, although many restoration projects focus primarily on re-establishing species-rich communities, there is growing interest in multifunctional outcomes of restoration (Manning et al. 2018) such as climate change mitigation or human livelihoods. For example, a project in Brazil’s Atlantic Forest that initially focused on the conservation of a highly endangered monkey species transformed into a landscape-scale restoration project seeking to simultaneously enhance the ecological integrity of the area as well as improving the food security of local communities (Chazdon et al. 2020). Sometimes, these new expectations can be controversial, because they can lead to simplistic restoration measures (e.g. indiscriminate planting of exotic trees). An analysis of restoration projects in 74 tropical countries revealed that a focus on social objectives translated into the use of the same few commercial and utilitarian species in many restoration initiatives, intensifying the homogenization of tropical ecosystems around the world (Martin et al. 2021). Both examples further underline the need for an integrative social–ecological framework to conceptualize restoration in a way that accounts for both ecological and social ambitions in restoration practice.

Restoration goals thus differ between restoration sites or projects, but can also change through time for a given site or project. While transparent goal-setting and monitoring should be a vital part of good restoration practice (Gann et al. 2019), we argue that restoration goals themselves also need to be considered in a dynamic way. For example, in some ecosystems, drying climatic conditions may require shifts towards different species assemblages than initially intended, or in parts of the Global South, societal demands might be intensifying such that restoration needs to more rapidly generate livelihood opportunities than initially recognized, while still safeguarding ecological sustainability in the long run.

In this paper, we propose a conceptual framework that can help scientists and decision-makers think about, communicate, and navigate changing restoration goals. The framework is based on observations by the author team of the dynamics of restoration projects with regard to ecological and social ambitions in already existing restoration projects. We term this framework the “social–ecological ladder of restoration ambition”, signalling that moving along this ladder can also help make restoration more integrative through time. Our framework is grounded in social–ecological systems thinking—that is, the application of systems thinking to interlinked social and ecological phenomena that involve dynamic feedbacks (Hobbs et al. 2011). With vast areas around the world being earmarked for restoration—but at the same time, with critique that some current restoration efforts are ecologically (Bond et al. 2019; Temperton et al. 2019) or socially (Löfqvist et al. 2022) short-sighted—it is important to offer pathways for restoration science and practice that encourage restoration to remain explicitly ambitious, open, and future-oriented (Higgs et al. 2018). Even if existing critiques of some large-scale restoration initiatives are warranted, perhaps these initiatives can still be seen as a valuable starting point. What is needed then is a way to think about how to further improve restoration outcomes, both ecologically and socially, in the longer term. Our paper seeks to provide a framework for this. We first outline our suggested framework and then illustrate it via two case studies representing a forest-dominated ecosystem in the Global South, and a grassland-dominated ecosystem in the Global North. In a final step, we discuss the general utility of applying the proposed framework.

## The social–ecological ladder of restoration ambition

Both ecological and social goals of restoration can be more or less ambitious. Ecological ambition is captured well by the “restorative continuum” (Gann et al. 2019), which depicts a gradient from reducing impacts, through remediation and rehabilitation of degraded systems, to fully recovering native ecosystems. Social ambition is more difficult to define because it is extremely multi-faceted. It might relate to the degree of stakeholder participation in the restoration process (see, for example, Arnstein’s (1969) classic “ladder of participation”), or more generally, to the material and non-material benefits that people obtain from restoration, including livelihoods (Erbaugh and Oldekop 2018), human-nature connectedness (Furness 2021), social cohesion (Alba-Patiño et al. 2021), or other dimensions of environmental justice (Löfqvist et al. 2022). In practice, the ecological and the social level of ambition are likely interrelated and as such can be captured by the notion of “social–ecological ambition”.

The level and type of ambition of any restoration project depend on the setting and actors involved (Carmenta and Vira 2018; Elias et al. 2022). Ambitions can change through time due to advancements in scientific knowledge (Perring et al. 2015), shifting stakeholder needs and values (Fox and Cundill 2018), alternating political orientation (Brunckhorst 2011), changing environments (Dudney et al. 2022), or the resources available to carry out restoration. In addition, past restoration outcomes can shape attitudes and expectations connected to future restoration projects (McGuire and Ehlinger 2022). These and other factors may lead to different modes of stakeholder participation, choices of focal species, or prioritized ecosystem functions and services. Pursuing fixed restoration goals therefore is too rigid in many instances—instead, goals need to be regularly re-assessed across both ecological and social realms. Arguably the best (though not always feasible) way to carry out restoration projects is to design interventions as active adaptive experiments (Keenleyside et al. 2012).

**Fig. 1:**
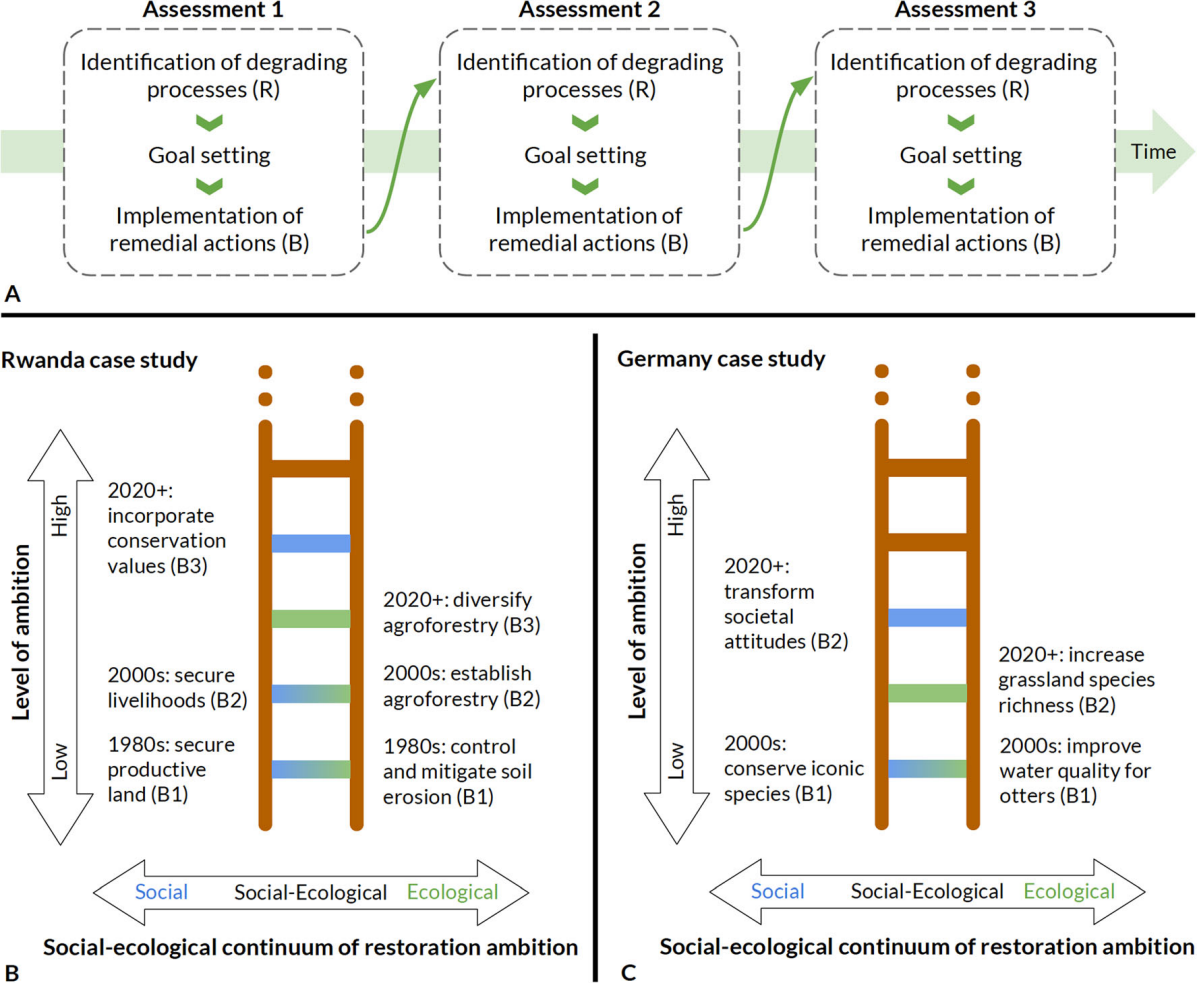
The social–ecological ladder of restoration ambition. Panel A: Restoration can follow three steps to replace degrading processes with restorative actions in an ecosystem or landscape: (1) assessment of the current social–ecological system state and identification of reinforcing feedback loops that drive degrading processes (R); (2) restoration goal setting; (3) implementation of remedial actions that disrupt or counteract the detrimental reinforcing feedback loop, or establish a balancing feedback loop instead (B). Steps 1–3 can be part of an active adaptive management approach and can be repeated as needs, values, environmental context and resources change over time. Panels B and C: Restoration actions take place within a social–ecological continuum, such that ambitious actions bridge social and ecological goals (purple rungs), signifying truly integrated social–ecological restoration. The text on the left and right of each ladder shows examples of the social and ecological ambitions that underpin the remedial actions that were undertaken in the study areas in Rwanda (Panel B) and Germany (Panel C). B1, B2 and B3 in brackets refer to the balancing feedback loops established through the remedial actions in each case study (please see the causal-loop diagrams of Figs. 3 and 4)

Three key concepts then are part of the social–ecological ladder of restoration ambition (Fig. 1). First, *degrading processes* relate to the drivers underpinning the need for restoration. In systems terms, degrading processes are typically reinforcing through time; that is, they are characterized by one or multiple reinforcing feedback loops. Reinforcing feedback loops, in fact, underpin the vast majority of contemporary sustainability problems and usually go hand-in-hand with exponentially increasing resource degradation (e.g. Steffen et al. 2011). Second, *restoration goals* are the ecological and social objectives to be achieved through restoration, as captured—for example—by quantifiable social and ecological indicators such as those in the restoration monitoring wheels proposed by the Society for Ecological Restoration (Gann et al. 2019) or the United Nations’ Food and Agriculture Organization (Buckingham et al. 2019). Third, *remedial actions* are the restoration interventions undertaken to reach a given restoration goal. In systems terms, remedial actions need to break the reinforcing feedback cycle characterizing degrading processes in order to “bend back” the curve of exponential resource degradation.

At any given point in time, then, a restoration project can be assessed—ideally using a formal adaptive management approach—with respect to its current restoration goal, the degrading processes that need to be stopped and reversed, and the remedial actions taken to move towards the desired goal. At a later point in time, the assessment can be repeated—ideally with a more ambitious goal than the first time around (Keenleyside et al. 2012), encouraging more multifunctional outcomes. Even if unintentional, such iterative goal-setting towards more and more ambitious levels of social–ecological restoration in fact characterizes many restoration projects, as illustrated by two case studies below.

## Case studies

**Fig. 2:**
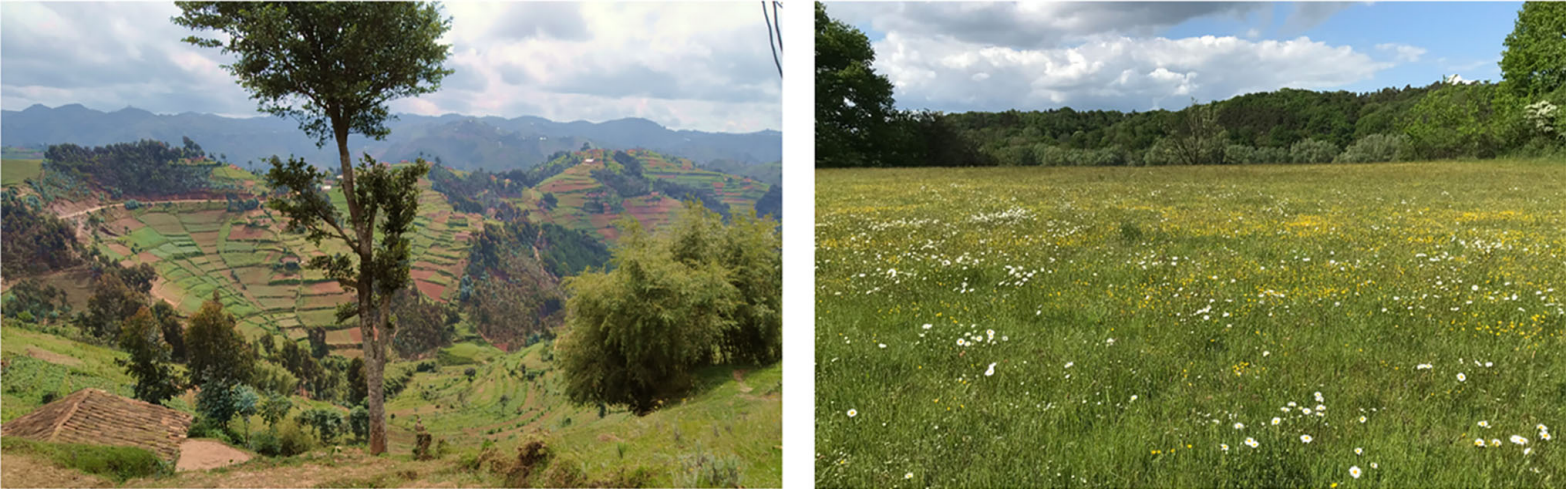
Photographs of the two case studies considered in this paper. Left: Forest landscape restoration in western Rwanda. Right: Grassland restoration in northern Germany. Photograph left: Joern Fischer. Photograph right: Konrad Gray

To illustrate our framework, we applied the social–ecological ladder of restoration ambition to two case studies. The case studies cover two very different social–ecological systems—one concerns forest landscape restoration in the Global South, the other grassland restoration in the Global North (Fig. 2). Despite major differences, both share conceptual commonalities in terms of changes in social–ecological restoration ambition through time. We present these case studies not as detailed empirical studies, but rather through stylized causal loop diagrams that capture, in qualitative and conceptual terms, the essence of key dynamics of social–ecological systems. We conceived both causal loop diagrams based on research experience on ecosystem restoration in Rwanda (BK, JF, MF) and Germany (MPR, VT, JF). Here, we focused on the most important system components that substantially shape dynamics in the respective restoration system (Haraldsson 2004).

### Case study I: Forest landscape restoration, Western Province, Rwanda

**Fig. 3:**
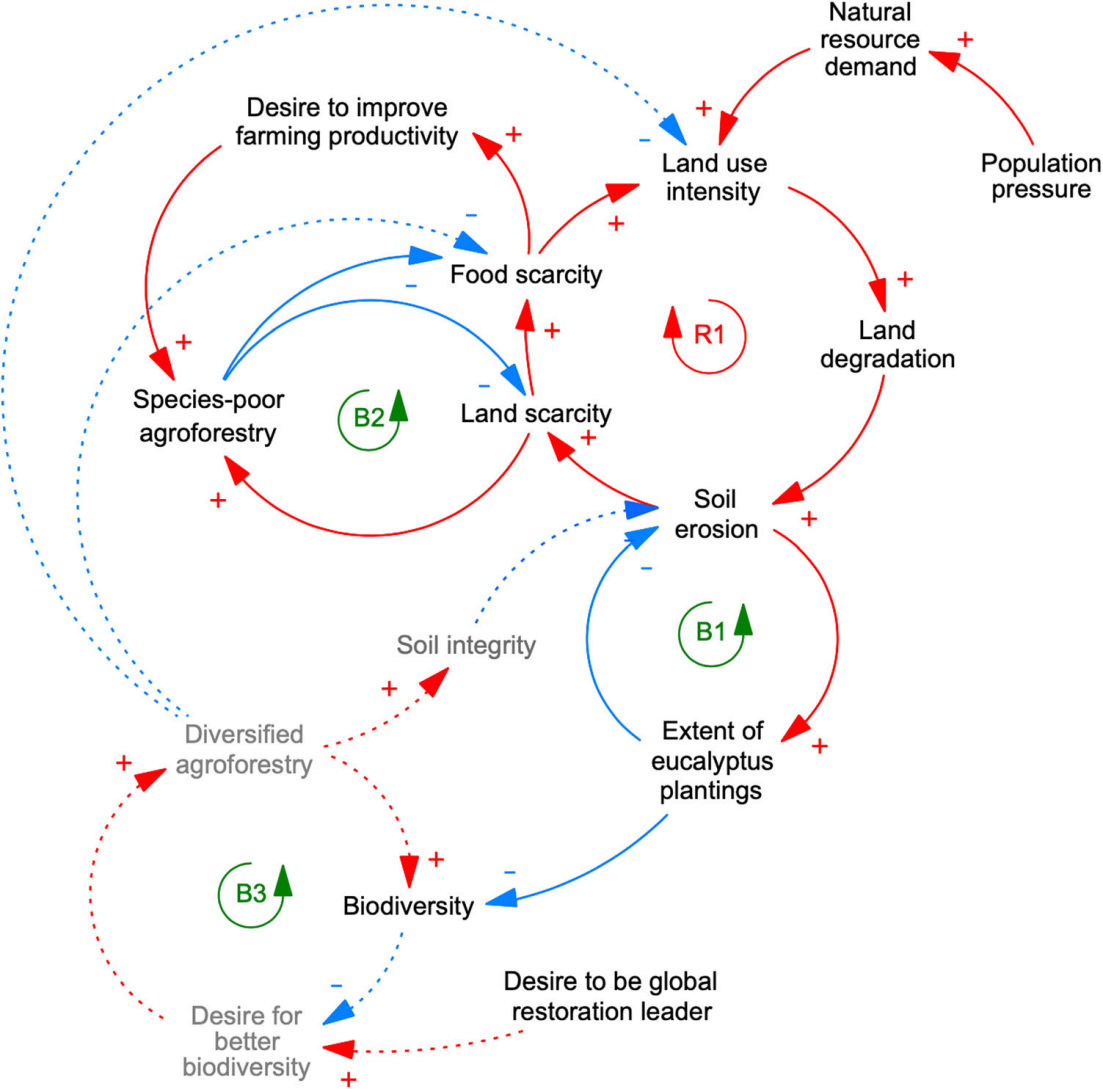
Causal-loop diagram of restoration dynamics in the Western Province of Rwanda. Restoration initially sought to reduce erosion (B1) and later specifically targeted land and food scarcity (B2), all of which were fuelled by high natural resource demand resulting in unsustainable land use (R1). In the future, the diversification of agroforestry (B3) could further contribute towards these restoration goals while also contributing to a more biodiverse landscape and healthier soils, moving towards the more ambitious end of the restorative continuum (sensu Gann et al. 2019). Variables printed in grey are possible future developments that are still in their early stages; dashed lines indicate possible future dynamics. Blue arrows with a “−” represent relationships between variables with a reducing effect (i.e. an increase in variable “a” leads to a decrease in variable “b”). Red arrows with a “ + ” indicate relationships with an enhancing effect (i.e. an increase in variable “a” leads to an increase in variable “b”). Closed cycles in the diagram indicate either balancing, self-regulating (B) or reinforcing, growing (R) feedback loops. Please note that causal loop diagrams only show the direction of an effect one system component has on another (i.e. reducing or enhancing effect) and not the extent of this effect

Rwanda has committed to restoring more than 80% of its terrestrial area by 2030 (IUCN 2020), and many restoration projects are underway throughout the country. Originally, modern restoration in Rwanda was motivated by large-scale degradation of ecosystems throughout the country caused by population pressure and excessive land use intensity in the 1970s (Nduwamungu 2011). In the Western Province, resource degradation was additionally exacerbated by commercialized tea production starting in the 1960s as well as an intensive dairy farming project in the 1980s (Clay 2019). Land was left depleted of native vegetation, resulting in soil erosion and landslides, which, in turn, increased land and food scarcity (Augenstein 2017) (initial degrading process characterized by reinforcing feedback loop R1 in Fig. 3).

As a response, fast-growing non-native plantation forests dominated by readily available and low-maintenance *Eucalyptus spp.* were established as of 1975 (first remedial action characterized by balancing feedback loop B1 in Fig. 3) (Arakwiye et al. 2021; Rwibasira et al. 2021). Through time, restoration more explicitly sought to address ongoing challenges of land and food scarcity and climate adaptation. Over the last two decades, the Rwandan government launched nation-wide programs aimed at economic and agricultural transformation (Government of Rwanda 2017; Weatherspoon et al. 2021; Kim et al. 2022). Associated measures to increase farming productivity such as crop intensification highlighted the need to counteract degrading processes such as soil erosion and led to both negative and positive environmental and societal impacts (Nyandwi et al. 2015; Isaacs et al. 2016; Clay 2018). Especially agroforestry was and continues to be highly promoted as part of Rwanda’s Vision 2020, with the goal of expanding to over 80% of agricultural land (second remedial action characterized by feedback loop B2 in Fig. 3).

Both remedial actions—monoculture woodlots and species-poor agroforestry—have provided short-term benefits for local communities such as fuelwood and more secure livelihoods. Yet, they have also resulted in a landscape with a patchy cover of mostly exotic trees, and agricultural plots with relatively low biodiversity (Arakwiye et al. 2021) and poor-quality soils (Rwibasira et al. 2021), whose resilience to climate change remains unclear. In terms of the restorative continuum (Gann et al. 2019), the interventions so far have mainly resulted in rehabilitation and reclamation, with little focus on more sophisticated semi-natural or native communities.

Today, rapid population growth and the legacy of past land-use decisions are fuelling ever-increasing pressure on natural resources throughout Rwanda. Hence, Rwanda is currently at a crossroads, and decisions on land-use and restoration practices will substantially influence the country’s future landscape. One possible trajectory is connected to the nation’s growing recognition of the importance of biodiversity and climate adaptation: complementing—and over time perhaps replacing—species-poor agroforestry with more diversified agroforestry could increase biodiversity and soil fertility, reduce soil erosion, and contribute to food security (possible future remedial action characterized by balancing feedback B3 in Fig. 3).

### Case study II: Grassland restoration, Lower Saxony, Germany

**Fig. 4:**
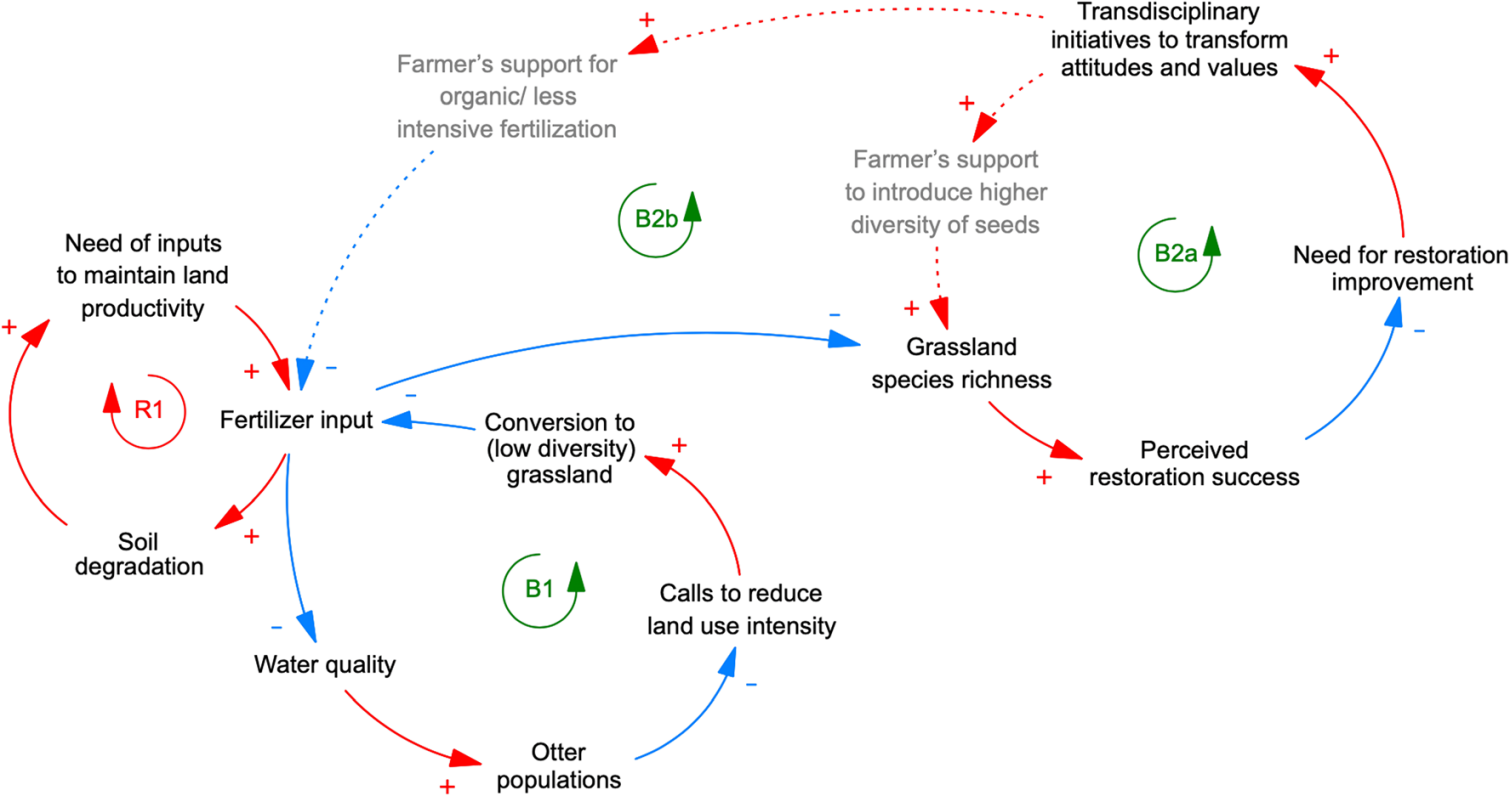
Causal-loop diagram of grassland restoration dynamics in Lower Saxony, Germany. Following degradation from intensive agriculture (R1), grassland restoration in the Ise floodplain in Lower Saxony was initially motivated by the desire to improve water quality and bring otters back to the river, with little focus on other aspects of biodiversity (B1). More recently, perceptions of low restoration success with respect to grassland diversity have sparked more ambitious restoration initiatives, including transdisciplinary collaborations that promote less intensive farmland management (B2a, b). Variables printed in grey are possible future developments that are still in their early stages; dashed lines indicate possible future dynamics. Blue arrows with a “-” represent relationships between variables with a reducing effect (i.e. an increase in variable “a” leads to a decrease in variable “b”). Red arrows with a “ + ” indicate relationships with an enhancing effect (i.e. an increase in variable “a” leads to an increase in variable “b”). Closed cycles in the diagram indicate either balancing, self-regulating (B) or reinforcing, growing (R) feedback loops. Please note that causal loop diagrams only show the direction of an effect one system component has on another (i.e. reducing or enhancing effect) and not the extent of this effect

Species-rich grasslands are among the most threatened ecosystems worldwide (Newbold et al. 2015), and their restoration is increasingly seen as important (Conrad and Tischew 2011). In Europe, the most prominent threats to grasslands are conversion to arable land, agricultural intensification (including agrochemical use) and land abandonment (Jacquemyn et al. 2011; Wesche et al. 2012). Since the 1950s, different regions in Germany have lost between 15 and 85% of species-rich grasslands (Wesche et al. 2012). Grassland restoration now constitutes one of the main compensation measures to counteract negative ecological impacts of infrastructure development (e.g. roads and railways) (Conrad and Tischew 2011). Most grassland restoration has taken place on former arable land (Conrad and Tischew 2011), where excessive fertilizer use typically causes declines in soil and water quality (i.e. degrading process characterized by a reinforcing feedback loop; R1 in Fig. 4).

In our case study (Fig. 4) near Gifhorn, Lower Saxony, the decrease of otter (*Lutra lutra*) populations in polluted waterways sparked calls to reduce land-use intensity. Some 20 years ago, one NGO began to convert arable lands into lower-intensity grasslands (first remedial action characterized by balancing feedback loop B1 in Fig. 4). This remedial action led to the recovery of otter populations, yet the biodiversity of the grasslands themselves remained low because of the use of low-diversity commercial seed mixtures. Many farmers also continued to apply relatively high levels of fertilizers (around 50 kg N per year per hectare) to the grasslands, which prevented their transition to more species-rich communities.

More recently, a value shift in what society perceives as successful grassland restoration has led to more ambitious restoration goals. New initiatives based on transdisciplinary approaches are now emerging that connect academia, NGOs, government institutions and farmers, and are beginning to foster a change in attitudes. These initiatives are testing and promoting less intensive management practices (e.g. lower fertilizer input) and more diverse seed mixtures to improve grassland species diversity (second remedial action characterized by balancing feedback loops B2a, b in Fig. 4).

## Two ways of using the ladder

These two case studies illustrate how the ladder of ambition can act as an analytical tool to identify and make sense of how degrading processes and remedial actions change within a restoration landscape as social and ecological ambitions shift. Ongoing social–ecological changes create a dynamic playing field in which restoration faces the challenge of responding to imminent threats such as erosion or decreases in a species’ population size, while also meeting social needs and values that change over time. The proposed framework acknowledges the co-occurrence and co-evolution of both social and ecological ambitions and offers a dynamic way of thinking about restoration planning. In practice, such a post hoc assessment of restoration processes informed by the “ladder way of thinking” would follow the three steps outlined above: (i) the assessment of the state of a social–ecological system at a given point in time and the identification of associated degrading processes, (ii) the identification of restoration goals at that point, and (iii) the identification of which remedial actions were implemented to counteract these degrading processes. In our case, we conducted expert interviews (as part of broader empirical projects), evaluated scientific publications and reports and designed causal loop diagrams for our two case studies, but additional methods such as original field data collection, participatory workshops or surveys may be required in other contexts. Figure 1 illustrates what the result of such an assessment looks like for the two case studies in Rwanda and Germany.

In addition to acting as an analytical tool, the ladder of ambition can also be applied to support decision-making in new or ongoing restoration projects. Drawing on adaptive management, the ladder of ambition can be used to design restoration interventions as active adaptive experiments from the start (Keenleyside et al. 2012), in order to test hypotheses on restoration outcomes in settings characterized by uncertainty (Allen and Gunderson 2011). In practice, this would entail the systematic assessment of the social–ecological context shaping a planned restoration project. Here, causal loop diagrams such as presented in Fig. 3 and Fig. 4 can provide a valuable overview of key variables and interactions that need to be considered (Meadows 2008). To design such causal loop diagrams, elements which have a significant influence on the system need to be identified, the relationships between these elements need to be understood, and the feedbacks between elements need to be analysed. This is best done in cooperation with diverse stakeholders with in-depth knowledge of the system in question (Haraldsson 2004). Next, based on such a detailed contextual understanding, future-oriented methods such as scenario planning or the three-horizons method (Sharpe et al. 2016) can help to define future restoration activities that account for both ecological and social ambitions of diverse groups of interest in a transdisciplinary way. Subsequent monitoring and evaluation of these activities, in turn, can generate valuable knowledge to inform iterative learning and guide the adjustment of actions and goals. This last step makes sure that restoration projects account for ongoing change in ambitions and is ideally guided by active adaptive management principles (Williams 2011)).

This combination of methods for (i) understanding the social–ecological context of restoration efforts (causal loop diagram), (ii) deciding on next steps for restoration activities in a transdisciplinary way (scenario panning, three horizons method), and (iii) iterative evaluation (active adaptive management) on the basis of integrated social–ecological thinking inspired by the ladder can help design long-term oriented restoration projects that consider both social and ecological ambitions and account for change. In other words, the ladder of ambition can act as a framework to support policymakers and practitioners to view restoration in an integrated way, while specific established methods help to translate this perspective into practice.

In summary, the two modes of application—analytical tool and decision-support tool—mean the ladder of ambition can benefit policymakers, practitioners and researchers at different stages of restoration projects. When using the ladder of ambition as an analytical tool, processes and ambitions in completed or ongoing restoration projects can be systematically assessed. This retrospective application generates a comprehensive understanding of the restored system that can provide insights for restoration in other contexts or inform the adaptation of ongoing activities. Used as a decision-making tool, the ladder of ambition can be applied before starting new restoration projects or as part of ongoing iterative processes. This proactive mode of application can inform future-oriented decisions by acknowledging the changing nature of ambitions instead of designing restoration interventions based on short-lived preferences.

Finally, the ladder of ambition could also be applied beyond restoration: diverse contexts in which social and ecological objectives coexist would also benefit from a more integrated way of approaching multiple ambitions that change over time. Examples range from biodiversity conservation to food security and the use of renewable resources. In all such instances, both ways of using the ladder could be valuable—as either an analytical tool for post hoc assessments or as decision-support tool.

## Implications for policy and practice

The ladder of ambition can help to navigate common challenges faced by restoration projects. Perhaps most importantly, contradictory social and ecological ambitions can imply trade-offs. When several desirable goals impair each other, actors are confronted with complex decisions regarding which ambitions to pursue to which degree. For example, a large-scale restoration project in Vietnam, while reaching its ecological goal of reforesting bare hills, led to unequal distribution of access to and benefits from forest resources (McElwee 2009). If and which trade-offs arise depends on the individual social–ecological context of a given system. By illustrating that (i) both social and ecological goals are important and (ii) goals change over time, the ladder of ambition can increase awareness of such trade-offs, as well as indicating ways to foster synergies through time.

Another common challenge in restoration relates to the question of whose ambitions are taken into account. Individual actors have different needs and values, leading to different visions for a given social–ecological system. In many cases, the people living in restored landscapes are not the ones who set restoration priorities due to power imbalances and lack of participation (Mansourian 2018). This can result in one-sided decisions that do not account for the diversity of ambitions actually present in a given system. For example, in Ghana, hierarchies in authority, control, and access over land-shaped decision-making, and excluded certain groups from participating in the design of a farmer-managed natural regeneration project (Kandel et al. 2021). Although power imbalances cannot be eradicated via a conceptual model, applying the ladder of ambition throughout a restoration process can help remind decision-makers that different ambitions likely coexist and may take precedence at different points in time of the restoration process.

Finally, stakeholders with a lot of decision-making power often prioritize short-term benefits over longer-term visions. In the context of restoration, such short-term thinking is problematic because restoration activities can take substantial time to yield beneficial results (Nerlekar and Veldman 2020), and some long-term pressures such as climate change require consideration now although their effects might not yet be visible. In the semi-arid and arid regions of China, for example, large-scale afforestation was motivated by the short-term goal to increase forest cover but did not adequately account for local environmental conditions, resulting in the planting of fast-growing yet ecologically inappropriate trees and shrubs which now impede restoration of native grasslands in the future (Cao et al. 2011). In such contexts, the ladder of ambition highlights the evolving nature of restoration goals and emphasizes the need for restoration to move beyond simple short-term fixes. This can sensitize decision-makers to consider ambitions for a given site for different points in time and to evaluate the feasibility and timing of diverse restoration options of different levels of ambition. In addition, instead of sticking to rigid, short-sighted goals that are set at the beginning of a restoration project, the ladder of ambition encourages iterative assessments of a given restoration project (see also Keenleyside et al. 2012).

Beyond addressing the specific challenges outlined above, the social–ecological ladder of restoration ambition contributes to improved restoration science and practice in three general ways. First, it suggests to view restoration sites as social–ecological systems, offering many of the benefits of thinking in systems (Meadows 2008). This includes the consideration of possible synergies and trade-offs between different ambitions, which increases the likelihood of identifying detrimental dynamics and fostering beneficial ones.

Second, establishing repeated re-assessments of restoration goals, processes, and remedial actions as part of restoration management makes restoration more adaptive and dynamic. This opens up room for making adjustments and prevents unsuitable trajectories to be continued just because they seemed appropriate in the past. Indeed, more routinely applying active adaptive management (and monitoring) provides important learning opportunities for restoration science and practice; starting with the rigorous assessment of social–ecological baseline conditions, the ladder of ambition is a way to think about successive layers of intertwined social and ecological restoration interventions.

Finally, restoration is inherently temporal: past land-use decisions cause a present need for restoration in social–ecological systems that will inevitably change in the future. The ladder of ambition integrates past, present and future through focusing on emerging possibilities rather than past deficiencies. It emphasizes that restoration is not a once-off effort but through time, can offer ever new opportunities to keep moving towards an increasingly more sustainable world.
